# Circadian disturbances and frailty risk in older adults: a prospective cohort study

**DOI:** 10.21203/rs.3.rs-2648399/v1

**Published:** 2023-03-27

**Authors:** Ruixue Cai, Lei Gao, Chenlu Gao, Lei Yu, Xi Zheng, David Bennett, Aron Buchman, Kun Hu, Peng Li

**Affiliations:** Brigham and Women’s Hospital; Brigham and Women’s Hospital; Brigham and Women’s Hospital; Rush University Medical Center; Brigham and Women’s Hospital; Rush University Medical Center; Rush University Medical Center; Brigham and Women’s Hospital; Brigham and Women’s Hospital/ Harvard Medical School

## Abstract

Frailty is characterized by diminished resilience to stressor events. It associates with adverse future health outcomes and impedes healthy aging. The circadian system orchestrates a ~24-h rhythm in bodily functions in synchrony with the day-night cycle, and disturbed circadian regulation plays an important role in many age-related health consequences. We investigated prospective associations of circadian disturbances with incident frailty in over 1,000 older adults who had been followed annually for up to 16 years. We found that decreased rhythm strength, reduced stability, or increased variation, were associated with a higher risk of incident frailty, and faster worsening of the overall frailty symptoms over time. Perturbed circadian rest-activity rhythms may be an early sign or risk factor for frailty in older adults.

## 1. Introduction

Frailty is defined as an age-related decline in multiple physiological systems.^1^ Frail older adults have increased vulnerability to stressor events, poorer quality of life,^2^ and increased risks for major adverse health outcomes, including Alzheimer’s disease.^3–6^ Frailty has emerged as a practical and unifying concept in the care of older people who experience multi-organ problems more commonly than a single-system illness.^1^ To inform appropriate interventions for preventing frailty incidence or its further progress and to promote successful aging, research is urgently needed to better understand the mechanisms of frailty. This study was designed to investigate the role of the circadian system as potential physiological correlates of frailty development.

Governed by the internal circadian clock, nearly all biological and physiological processes in humans, such as sleep and motor activity, show ~ 24-h rhythms as an evolutional adaptation to the daily environmental changes.^7^ Disrupted circadian control leads to altered circadian rhythms in physiological processes or daily behaviors, as observed with aging and in neurodegenerative diseases.^8^ For example, compared to younger adults, older people have suppressed circadian rest-activity rhythms with advanced phase;^9^ and these circadian changes were further degraded with aging within the same older adults.^10^ Additionally, changes in circadian rest-activity rhythms have been linked to the future development of many chronic diseases, such as type 2 diabetes, Alzheimer’s disease, and Parkinson’s disease.^10–12^ Given the high prevalence of both circadian dysfunction and frailty in older adults,^13^ establishing their link is important but awaits more systematic studies.

The goal of the current study is to determine the relevance of circadian disturbances to incident frailty and the change in frailty symptoms over time in older adults. We hypothesized that older adults with more perturbed circadian rest-activity rhythms were at increased risk for incident frailty and had a faster deterioration in frailty symptoms.

## 2. Methods

### 2.1 Study design

We studied participants in the Rush Memory and Aging Project (MAP)^14^, an ongoing prospective, observational cohort study conducted at the Rush Alzheimer’s Disease Center, Rush University Medical Center. The MAP started in 1997, and over 2,100 participants have agreed to receive annual clinical evaluations, cognitive and frailty assessments. Starting in 2005, actigraphy (i.e., collection of daily motor activity data with an omnidirectional accelerometer) was added to the annual visit in the MAP. In the current study, the visit when the first actigraphy assessment was performed was used as the analytic baseline for each participant. Using the daily motor activity data, we evaluated baseline circadian rest activity rhythms and linked the results to incident frailty and changes in frailty symptoms during follow-up visits. The MAP was approved by an Institutional Review Board of Rush University Medical Center. All participants signed an informed consent and a repository consent to allow their data to be shared with broader research community. The current study was reviewed and approved by the Institutional Review Board of Mass General Brigham.

### 2.2 Participants

**Figure 1:**
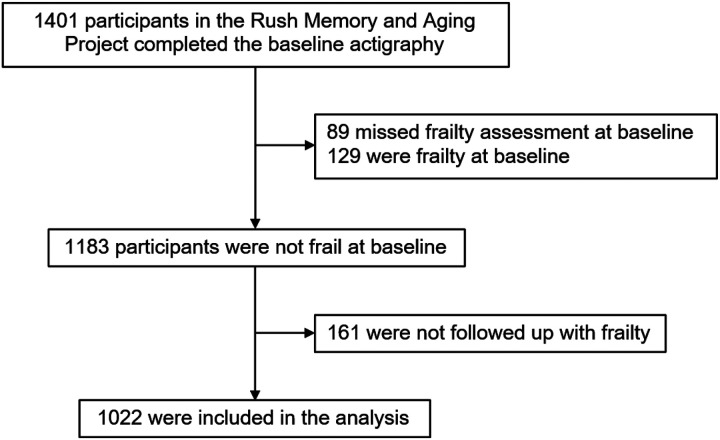
Flow of participants through the study

A total of 1,401 participants completed baseline actigraphy assessment. Among them, 218 participants were frail or did not have frailty assessment at baseline actigraphy assessment, and they were excluded. After that, 161 participants who had no follow-up frailty assessments were further excluded. This resulted in 1,022 participants who entered the subsequent analyses (Fig. 1). By May 2022, they had been followed for up to 16 years at the time the data were frozen for these analyses (range: 1–16 years; mean: 5.9 years; standard deviation: 3.8 years).

### 2.3 Assessment of circadian rest-activity rhythms

Participants wore the Actical device (Philips Respironics, Bend, OR, USA) on their non-dominant wrist for up to 14 days (range: 7–14 days). Raw data (i.e., 3-dimensional accelerometer data) from the Actical device were sampled at 32 Hz and were integrated into 15-second epochs (i.e., activity counts). The activity counts recordings were subject to signal quality screenings with the assistance of an established MATLAB GUI program (Ver. R2015a, the MathWorks Inc., Natick, MA, USA).^15,16^ Quality issues such as 1) isolated huge spikes with amplitude going beyond 10 standard deviations away from the individual global mean levels; and 2) sequences of zeros with duration > 60 minutes during the daytime (likely occurred when subjects took the device off) were identified and marked as gaps.^17^ These gaps were treated as missing data in subsequent analyses.

To estimate circadian regulation/disturbance, we extracted the rest-activity rhythms of ~ 24-h from actigraphy recordings using the uniform phase empirical mode decomposition (UP-EMD) analysis.^18^ Unlike the traditional cosinor analysis, the UP-EMD analysis does not assume stationary oscillatory components in a signal (i.e., each rhythm is a sine/cosine wave with a constant amplitude and a fixed cycle length) and thus, it can better extract the biological rhythms that are oftentimes nonstationary (i.e., with varying amplitudes and cycle lengths) and nonlinear (i.e., non-sine/cosine waveforms). Using the UP-EMD-derived oscillatory component with a ~ 24-h cycle length, three rhythmicity measures were derived: (1) The amplitude was calculated as the absolute value of the Hilbert transform of the UP-EMD extracted ~ 24-h oscillatory component, which represents the strength of the rhythm. To alleviate the influence of individual activity level for a fair between-participant comparison, the amplitude was normalized by the individual standard deviation to have a normalized strength of the rhythm. (2) The acrophase which is the mean of the phase marker for peak timing across all ~ 24-h cycles in the oscillatory component was used to quantify the timing of peak activity level in a day. (3) The variation of cycle length was calculated as the standard deviation of all cycle lengths from the UP-EMD extracted oscillatory component. To be consistent across all participants, the above calculations were implemented to the first six cycles of the extracted oscillatory component, and the corresponding participant would be excluded if the oscillatory component had less than six cycles. For comparison purposes, the 24-h amplitude and acrophase of daily rest-activity rhythms were also calculated based on the traditional cosinor analysis.^19^

In addition, a set of nonparametric analyses were used to obtain the following rest-activity rhythm metrics: interdaily stability (IS) was used to quantify the day-to-day robustness of the 24-h activity patterns (i.e., a greater IS indicates more regular 24-h rhythm); intradaily variability (IV) was used to quantify the fragmentation of the 24-h activity patterns (i.e., a greater IV indicates more fragmented rest-activity rhythm); the average activity level during the most active 10-h period (M10) was calculated to represent physical activity intensity during the active period (usually when awake; a higher M10 indicates higher activity intensity); the average activity level during the least active 5-h period (L5) was calculated to represent the level of restfulness during the inactive period (usually during sleep; a lower L5 represents more restfulness); and relative amplitude ($\text{RA}=\frac{M10-L5}{M10+L5}$) was used to estimate the strength of the 24-h rest-active rhythm. RA was categorized into low and high according to the median to correct the left-skewness.

### 2.4 Assessment of frailty

Frailty was assessed annually based on five components,^3,20^ including grip strength, gait speed, body mass index (BMI), fatigue, and physical activity. Grip strength was measured using the Jamar hydraulic hand dynamometer (Lafayette Instruments) and was the average readout across four trials (two per hand). Gait speed was based on the time to walk eight feet. BMI was calculated as weight divided by height (kg/m^2^). Fatigue was assessed using two questions derived from a modified version of the Center for Epidemiologic Studies-Depression Scale (CES-D): (1) I felt that everything I did was an effort, and (2) I could not get “going”. Self-reported physical activity was based on the number of hours per week that participants engage in five types of activities: walking, gardening, calisthenics, bicycle riding, and swimming.

The scores in the lowest quintile of gait speed, grip strength, BMI, and physical activity, and a response of “yes” to one or both questions for fatigue, were considered consistent with frailty.^3^ Due to level differences in performance measures between men and women, sex-specific quintiles were used for grip strength, gait speed, and physical activity. Similar to the Fried Frailty Phenotype,^21^ frailty was defined as the presence of 3 or more components.

In addition, a continuous composite score of frailty^3,22^ were also computed based on grip strength, gait speed, BMI, and fatigue. The continuous frailty score was constructed by averaging the z scores of the four components, which were calculated based on the means and standard deviations from the whole cohort at baseline. For this frailty score, a greater value means that a participant is frailer. Grip strength and BMI were multiplied by −1 so that larger values reflected poorer performance.

### 2.5 Covariates

We considered the following covariates: age, sex, years of education, sleep duration, sleep fragmentation, vascular disease burden, and vascular disease risk. Sleep duration was calculated by the total nighttime sleep hours based on actigraphy data. Sleep fragmentation was estimated by a prior established metric based on actigraphy that estimates the transition probabilities of rest-to-activity states.^23^ A greater value represents more fragmented sleep. Vascular disease burden was estimated as the sum of self-reported claudication, stroke, heart conditions, and congestive heart failure.^24^ Vascular risk was evaluated by a sum of self-reported hypertension, diabetes, and smoking history.^25^

### 2.6 Statistical analysis

We performed three types of statistical analyses. (1) Cox proportional hazards models were used to test the associations of disturbances in circadian rest-activity rhythms with incident frailty. In each of the primary models, one of the rhythmicity measures (amplitude, acrophase, variation of cycle length, IS, IV, M10, or L5) was included as a predictor while controlling for age, sex, and education. Additional models were performed by adding sleep duration, sleep fragmentation, vascular disease burden, and vascular disease risk as additional covariates to the primary models. As secondary analyses, we performed similar cox proportional hazards models for 24-h amplitude, 24-h acrophase, and RA. We also determined the individual physical activity levels based on actigraphy-derived total daily activity. Since physical activity (self-report) is one component of frailty which was collinear with the actigraphy-derived total daily activity and rhythmicity measures, to examine whether any observed associations may be confounded by total activity, we repeated the cox proportional hazards models in participants with relatively lower total daily levels (i.e., lower than the cohort median). (2) Linear mixed-effects models were used to test the associations of rest-activity rhythmicity measures at baseline with the longitudinal change in the continuous frailty score. In each of the primary models, the frailty score was included as a continuous dependent variable, and the time since baseline (in years) and a specific metric of circadian rest-activity rhythms, and their interaction as fixed-effect predictors. In all these models, participant and time since baseline were included as random-effects predictors. Age, sex, and education were adjusted, and items for their interactions with time since baseline were included. The potential effects of, sleep duration, sleep fragmentation, vascular disease burden, vascular disease risk factor, and their interactions with time were further considered and controlled. (3) To explore whether the associations specifically exist in each component of frailty, the same linear mixed-effects models were repeated for three of the four frailty components (i.e., grip strength, gait speed, BMI), and for fatigue which is a categorical outcome with three levels, we used generalized mixed models with a random intercept and a cumulative logit link. All statistical analyses were performed in R (version 4.1.2). Statistical significance was considered at two-tailed alpha level of 0.05.

## 3. Results

### 3.1 Demographics and clinical characteristics

Demographics and clinical characteristics of the 1,022 participants at analytical baseline are summarized in Table 1. The mean age at baseline was 81 years old (standard deviation: 7.2; range: 59–100 years old). Among them, 74.6% were female.

### 3.2 Circadian rest-activity rhythms and incident frailty

**Figure 2:**
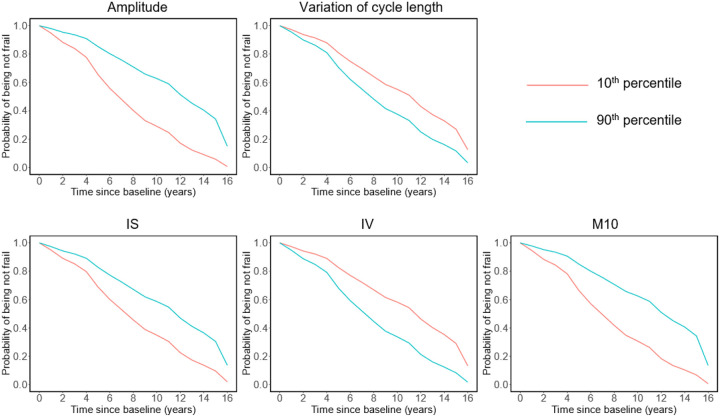
Predicted survival curves from Cox proportional hazards models. The predicted probabilities of being not frail for two representative participants with circadian metrics amplitude, variation of cycle length, IS, IV, or M10 at the 10^th^ and 90^th^ percentiles, respectively. Abbreviation: IS, interdaily stability; IV, intradaily variability; M10, the average activity during the most active 10-h.

Correlations of circadian rest-activity metrics at baseline are summarized in Figure S1. Over a mean of 5.9 years of follow-up, 357 (34.9% of 1,022) participants developed frailty. Older age at baseline, female sex, and shorter education years were associated with increased risk for frailty (Table 2). After adjusting for age, sex, and education, higher risk of frailty was observed in participants with reduced amplitude, increased variation of cycle length, reduced IS, increased IV, and reduced M10. The hazard ratios (HR) of frailty corresponding to each 1-SD change in these metrics ranged from 1.29 to 1.48 with all *ps* < 0.001 (Table 2). Figure 2 shows the probability of being not frail for two participants with the amplitude, variation of cycle length, IS, IV, and M10 at the 10th (low) and 90th percentile (high) in this cohort. All these associations remained statistically significant with slightly attenuated HR after further adjustments of sleep duration, sleep fragmentation index, vascular disease burden, and vascular risk factors (Table S1). Consistently, reduced 24-h amplitude (calculated using the traditional cosinor analysis) and low RA (calculated based on the nonparametric analysis) were also associated with incident frailty after adjustment for age, sex, and education (Table S2). The timing of the rhythm (either acrophase calculated from UP-EMD or the 24-h acrophase from cosinor analysis) and L5 were not associated with incident frailty (Table 2, Table S2).

### 3.3 Circadian rest-activity rhythms and change in frailty score

**Figure 3:**
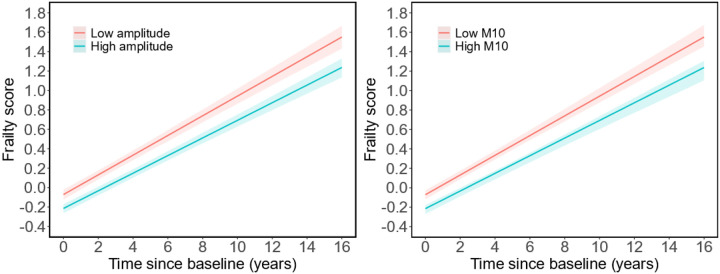
Predicted trajectory of frailty score. The predicted frailty score increases for two representative participants with low amplitude or M10 (the 10^th^ percentile), and high amplitude or M10 (the 90^th^ percentile). Abbreviation: M10, the average activity during the most active 10-h.

Frailty score increased over time with an average annual increase of 0.095 unit (standard error [SE]: 0.002; p < 0.001), and the annual increase was accelerated by 0.002 unit (SE: 0.0003) when a participant was one year older than the cohort mean at baseline (p < 0.001). After adjusting for age, sex, and education, the increase in the frailty score was much faster in participants with smaller amplitude or smaller M10, i.e., for 1-SD decrease in amplitude or M10, the annual increase was accelerated by 0.004 ± 0.002 (*p* = 0.047) or 0.005 ± 0.002 (*p* = 0.011) (Table 3). These effects were equivalent to that of being 2 to 2.5 years older than the cohort mean age at baseline. To illustrate the association between baseline amplitude, M10 and the rate of increase in frailty score, the predicted trajectories of the continuous composite frailty score for different levels of amplitude and M10 are shown in Fig. 3. Specifically, when compared to the high (i.e., the 90th percentile) level, participants in low level (i.e., the 10th percentile) of amplitude or M10 exhibited a steeper increase in frailty score over 16 years of follow-up. These associations persisted after controlling for sleep duration, sleep fragmentation, vascular disease burden, vascular risk factors, and their interactions with time (Table S4).

### 3.4 Circadian rest-activity rhythms and change in individual components of frailty

We used similar mixed models to test the associations of baseline circadian metrics with the rate of change in each of four frailty components separately (Table S5). A more rapid decline in grip strength was associated with smaller IS, greater IV, and smaller M10 after adjusting for age, sex, education, and their interactions with time. Specifically, for 1-SD decrease in IS or M10, or 1-SD increase in IV, the annual decrease in grip strength was accelerated by 0.119±0.045 (*p*=0.008) or 0.107±0.045 (*p*=0.019), or 0.142±0.049 (*p*=0.004), which were equivalent to the effect of being 4 to 5 years older than the cohort mean at baseline (note that the average annual rate of decrease in grip strength was around 1.650, and being one year older was associated with an extra decrease of 0.024 ~ 0.029 per year in that three models). The association of smaller IS, and greater IV with grip strength was statistically significant after further controlling for sleep duration, sleep fragmentation, vascular disease burden, vascular risk factors, and their interactions with time. None of the circadian metrics was associated with the rate of change in gait speed. A rapid decline in BMI was observed in those with smaller amplitude, smaller IS, and smaller M10 after adjusting for demographics and their interactions with time. Specifically, for each 1-SD decrease in IS, the annual decrease in BMI was accelerated by 0.037±0.015 (*p*=0.012), which was equivalent to an accelerated decline for being 6 years older at baseline (note that the average annual rate of decrease in BMI was 0.117, and being one year older at baseline was linked to an extra decrease of 0.006 in BMI per year); for each 1-SD decrease in amplitude or M10, the annual decrease in BMI was accelerated by 0.053±0.015 (p<0.001) or 0.043±0.015 (p=0.004). These associations remained statistically significant after further controlling for other covariates. After adjusting for demographics and their interactions with time (note that baseline age was not associated with the annual change in fatigue score), worsening of fatigue over time was much faster in subjects with smaller acrophase, smaller M10 and smaller L5, i.e., for each 1-SD decrease in acrophase, or M10, or L5, the annual increase in fatigue score was accelerated by 0.020±0.010 (*p*=0.035), or 0.021±0.009 (*p*=0.028), or 0.019±0.008 (*p*=0.023). These associations were not significant after further controlling for other covariates.

## 4. Discussion

By analyzing data from a cohort of over 1,000 older adults, we found that disturbances in circadian rest-activity rhythms, especially reduced rhythm strength, reduced stability, and increased variation of cycle length, were associated with increased risk for frailty and faster worsening of frailty symptoms, in particular, the decrease in grip strength, reduction of BMI, and increasing fatigue. It is known that circadian function plays an important role in many age-related diseases, such as cardiometabolic disorders,^26^ cognitive impairment,^27^ and Alzheimer’s disease.^10^ Our new results add a significant pillar to this important role of circadian function in aging by showing that it associates to the risk of developing frailty in future.

In the current study, the average time lag between baseline actigraphy assessments and the incidence of frailty is 5.9 years a relatively long window with detectable changes in circadian rest-activity rhythms prior to the development of frailty, suggesting a potential of these actigraphy-based measures as early indicators of future frailty. Alternatively, the disturbances in circadian rest-activity rhythms may directly pose a risk of developing frailty on older adults. Future studies are thus warranted to untangle this potentially causal relationship, and if confirmed, strategies such as life-style interventions to consolidate the rest-activity rhythms may be applied as a proactive way to prevent older adults from developing frailty.

We note that the association between lower circadian amplitude and incident frailty may be explained by either reduced peak activity during daytime, or increased activity nadir during nighttime, or both. Our results showed that the association of incident frailty with M10, a measure of daytime activity level, was statistically significant whereas it was not with L5, a measure of nighttime activity level. Together with the fact that the circadian metrics were extracted from the profile of daily motor activity, it is possible that reduced daytime activity or exercise, a known risk factor of frailty,^28^ may confound our observations. Since physical activity is one component of the frailty phenotype and is collinear with actigraphy-derived circadian measures, it is not appropriate for us to adjust for it in statistical models. To elucidate their relationships, we performed a set of sensitivity analyses by including only participants who had relative lower physical activity. The results showed that the observed associations of incident frailty with rest-activity rhythm measures (amplitude, variation of cycle length, IS, and M10, except for IV) remained statistically significant (Table S3), suggesting that the relationships between these circadian measures and frailty are independent from that of physical activity.

In a previous cross-sectional study of 105 older community-dwelling subjects,^29^ an association between RA and frailty was reported. However, in another cross-sectional study among 69 institutionalized older adults,^30^ the associations between rest-activity rhythms and frailty were not significant. The discrepancy between these two studies may be attributable to differences in study population, frailty assessment, and proportion of frail participants. In addition, the cross-sectional design and the relatively small sample size in the two studies might complicate the interpretation of results. Using a longitudinal design and a much larger sample size (> 1,000), this study provided convincing evidence for the link between rest-activity rhythms and the development of frailty.

Previous studies have suggested that frailty is a dynamic process, i.e., within a short term, the severity of frailty may fluctuate but it tends to be worsening within each individual over a longer term.^3,31,32^ To document change in the degree of frailty over time, we used a continuous composite measure of frailty based on the four components^3^. We found that smaller IS, greater IV, and smaller M10 were associated with a more rapid decline in grip strength, and that smaller amplitude, smaller IS, and smaller M10 were associated with a more rapid decline in BMI. Smaller acrophase, M10, and L5 were also associated with a faster increase in fatigue. Previous studies showed frailty components may have a different sequence of emergence before the onset of frailty.^33^ Our results demonstrate that circadian rest-activity rhythms may be sensitive to changes in individual frailty components and have the potential to be used in identifying individuals at risk for frailty at early stages.

There are many possible pathways through which circadian disturbances may lead to a higher risk of frailty. First, circadian rhythms may affect frailty through disrupted sleep. Disturbed circadian rhythms have been linked to various sleep problems or disorders, such as insomnia, poor sleep quality, and reduced sleep duration, which have been reported as predictors of frailty.^34,35^ Second, disturbed circadian rest-activity rhythms have also been linked previously to alterations of metabolism^11^ and inflammatory markers^36^ in older adults. Since metabolic function and inflammation are considered of key importance in frailty,^37^ circadian disturbances may contribute to increased risk of frailty through impaired metabolism and enhanced inflammation. Finally, circadian disturbances may lead to oxidative damage and neuronal loss in the brain.^38,39^ Several studies have shown that aging-related changes in the brain were associated with frailty.^32,40,41^ Therefore, circadian disturbances may contribute to the development of frailty through brain aging. Growing studies suggest a possible bidirectional relationship between cognitive impairment and frailty,^32,42^ in which the concept of cognitive frailty was proposed.^43^ Together with our prior findings, the results provide evidence for circadian disturbance may be one of common pathological mechanisms for cognitive impairment and frailty. Future studies should be conducted to elucidate the relationship between circadian rhythms and cognitive frailty.

Strengths of our study include the longitudinal design, long duration of follow-up and comprehensive measures of circadian rest-activity rhythms. Our study also has several limitations. The participants had a mean age of more than 80 years at baseline, caution should be taken when translating our findings to younger populations. In addition, it is known that rest-activity rhythms derived from actigraphy data are easily masked by schedules and the environmental conditions. Further studies on the associations between the endogenous circadian function and frailty are warranted.

With advances in wearable technology, actigraphy-based assessment of circadian daily rhythms can be a simple tool for long-term health monitoring in older adults. Along with other clinical and physiological measures, actigraphy-based circadian measures may improve the identification of older adults at risk of frailty, making it possible for these individuals to benefit from early interventions (e.g., life-style intervention, nutritional supplementation, etc.)^44^ Whether improving circadian rhythms using behavioral interventions or light treatment^45,46^ can help prevent or slow the development of frailty merits further investigation.

## Figures and Tables

**Table 1:** Demographic and clinical characteristics of participant at baseline

Variable	Participants (n = 1022) Mean (SD) or N (%)
Age (year)	81.0 (7.2)
Female	762 (74.6%)
Education (year)	15.2 (3.0)
Depression symptoms	0.82 (1.34)
Sleep duration	4.92 (1.46)
Sleep fragmentation	0.03 (0.01)
Motor function	1.03 (0.22)
Vascular disease burden	0.35 (0.66)
Vascular risk factors	1.10 (0.80)
Circadian rhythmicity characteristics	
Amplitude	0.34 (0.10)
Acrophase	13.14 (1.53)
Variation of cycle length	1.36 (0.91)
IS	0.52 (0.12)
IV	1.17 (0.27)
M10	18172.70 (10305.50)
L5	1591.60 (1640.88)
Frailty score	−0.11 (0.49)
Grip strength	46.65 (17.58)
Gait speed	4.43 (1.77)
BMI	27.25 (5.25)
Fatigue	0.32 (0.55)
Physical activity	3.72 (3.73)

Abbreviations: SD, standard deviation; IS, interdaily stability; IV, intradaily variability; M10, the average activity during the most active 10-h; L5, the average activity during the least active 5-h period; RA, relative amplitude; BMI, body-mass index.

**Table 2:** Association of circadian metrics and incident frailty after adjustment for demographics

	Amplitude	Acrophase	Variation of cycle length	IS	IV	M10	L5
	HR (95%CI) *p* value	HR (95%CI) *p* value	HR (95%CI) *p* value	HR (95%CI) *p* value	HR (95%CI) *p* value	HR (95%CI) *p* value	HR (95%CI) *p* value
Age^a^	1.10 (1.08–1.13) < 0.001	1.11 (1.09–1.13) < 0.001	1.11 (1.09–1.13) < 0.001	1.12 (1.09–1.14) < 0.001	1.10 (1.08–1.13) < 0.001	1.10 (1.08–1.12) < 0.001	1.11 (1.09–1.13) < 0.001
Female sex	3.47 (2.44–4.92) < 0.001	3.02 (2.13–4.26) < 0.001	3.40 (2.38–4.85) < 0.001	3.34 (2.36–4.74) < 0.001	3.38 (2.38–4.80) < 0.001	3.38 (2.39–4.79) < 0.001	3.14 (2.22–4.45) < 0.001
Education^b^	1.07 (1.03–1.12) 0.001	1.06 (1.01–1.10) 0.008	1.06 (1.02–1.11) 0.003	1.07 (1.02–1.11) 0.002	1.06 (1.02–1.11) 0.002	1.06 (1.02–1.10) 0.004	1.05 (1.01–1.09) 0.013
Amplitude^c^	1.48 (1.31–1.67) < 0.001						
Acrophase^c^		1.01 (0.89–1.13) 0.925					
Variation of cycle length^d^			1.29 (1.16–1.44) < 0.001				
IS^c^				1.30 (1.16–1.46) < 0.001			
IV^d^					1.31 (1.16–1.49) < 0.001		
M10^c^						1.47 (1.29–1.68) < 0.001	
L5^d^							1.08 (0.97–1.21) 0.146

a Results for 1-unit increase,

b Results for 1-unit decrease,

c Results for 1-SD decrease,

d Results for 1-SD increase.

Abbreviation: SD, standard deviation; HR, hazard ratio; CI, confidence interval; IS, interdaily stability; IV, intradaily variability; M10, the average activity during the most active 10-h; L5, the average activity during the least active 5-h period.

**Table 3:** Circadian metrics and change in frailty after adjustment for demographics

	Amplitude	Acrophase	Variation of cycle length	IS	IV	M10	L5
	Estimate (SE) *p* value	Estimate (SE) *p* value	Estimate (SE) *p* value	Estimate (SE) *p* value	Estimate (SE) *p* value	Estimate (SE) *p* value	Estimate (SE) *p* value
Intercept	−0.142 (0.017) < 0.001	−0.147 (0.017) < 0.001	−0.142 (0.017) < 0.001	−0.141 (0.017) < 0.001	−0.140 (0.017) < 0.001	−0.145 (0.016) < 0.001	−0.146 (0.017) < 0.001
Time	0.096 (0.003) < 0.001	0.095 (0.002) < 0.001	0.095 (0.003) < 0.001	0.095 (0.003) < 0.001	0.096 (0.003) < 0.001	0.096 (0.003) < 0.001	0.095 (0.002) < 0.001
Age	0.031 (0.002) < 0.001	0.033 (0.002) < 0.001	0.033 (0.002) < 0.001	0.033 (0.002) < 0.001	0.030 (0.002) < 0.001	0.030 (0.002) < 0.001	0.032 (0.002) < 0.001
Age · time	0.002 (0.0003) < 0.001	0.002 (0.0003) < 0.001	0.002 (0.0003) < 0.001	0.002 (0.0003) < 0.001	0.002 (0.0003) < 0.001	0.002 (0.0003) < 0.001	0.002 (0.0003) < 0.001
Sex (male)	−0.052 (0.034) 0.130	−0.030 (0.034) 0.374	−0.048 (0.034) 0.161	−0.049 (0.034) 0.156	−0.056 (0.034) 0.101	−0.044 (0.034) 0.189	−0.036 (0.034) 0.295
Sex (male) · time	−0.007 (0.005) 0.145	−0.007 (0.005) 0.165	−0.006 (0.005) 0.268	−0.007 (0.005) 0.172	−0.007 (0.005) 0.160	−0.007 (0.005) 0.152	−0.006 (0.005) 0.211
Education	−0.006 (0.005) 0.264	−0.003 (0.005) 0.520	−0.004 (0.005) 0.446	−0.005 (0.005) 0.347	−0.006 (0.005) 0.242	−0.006 (0.005) 0.220	−0.004 (0.005) 0.375
Education · time	0.0001 (0.001) 0.933	0.00002 (0.001) 0.788	0.0003 (0.001) 0.709	0.0002 (0.001) 0.787	0.0001 (0.001) 0.850	0.0004 (0.001) 0.638	0.0004 (0.001) 0.554
Amplitude^a^	−0.058 (0.015) < 0.001						
Amplitude · time^b^	−0.004 (0.002) 0.047						
Acrophase^a^		0.033 (0.015) 0.026					
Acrophase · time^b^		−0.004 (0.002) 0.077					
Variation of cycle length^a^			0.040 (0.015) 0.007				
Variation of cycle length · time^b^			−0.002 (0.002) 0.301				
IS^a^				−0.038 (0.015) 0.011			
IS · time^b^				−0.001 (0.002) 0.603			
IV^a^					0.061 (0.015) < 0.001		
IV · time^b^					0.002 (0.002) 0.290		
M10^a^						−0.062 (0.015) < 0.001	
M10 · time^b^						−0.005 (0.002) 0.011	
L5^a^							−0.010 (0.014) 0.486
L5 · time^b^							−0.002 (0.002) 0.258

a Results for 1-SD change,

b Results for 1-SD · 1-year change.

Abbreviation: SD, standard deviation; SE, standard error; IS, interdaily stability; IV, intradaily variability; M10, the average activity during the most active 10-h; L5, the average activity during the least active 5-h period.
